# The pairwise disconnectivity index as a new metric for the topological analysis of regulatory networks

**DOI:** 10.1186/1471-2105-9-227

**Published:** 2008-05-02

**Authors:** Anatolij P Potapov, Björn Goemann, Edgar Wingender

**Affiliations:** 1 Department of Bioinformatics, Medical School, Georg August University of Göttingen, Goldschmidtstrasse 1, D-37077 Göttingen, Germany; 2 BIOBASE GmbH, Halchtersche Strasse 33, D-38304 Wolfenbüttel, Germany

## Abstract

**Background:**

Currently, there is a gap between purely theoretical studies of the topology of large bioregulatory networks and the practical traditions and interests of experimentalists. While the theoretical approaches emphasize the global characterization of regulatory systems, the practical approaches focus on the role of distinct molecules and genes in regulation. To bridge the gap between these opposite approaches, one needs to combine 'general' with 'particular' properties and translate abstract topological features of large systems into testable functional characteristics of individual components. Here, we propose a new topological parameter – the pairwise disconnectivity index of a network's element – that is capable of such bridging.

**Results:**

The pairwise disconnectivity index quantifies how crucial an individual element is for sustaining the communication ability between connected pairs of vertices in a network that is displayed as a directed graph. Such an element might be a vertex (i.e., molecules, genes), an edge (i.e., reactions, interactions), as well as a group of vertices and/or edges. The index can be viewed as a measure of topological redundancy of regulatory paths which connect different parts of a given network and as a measure of sensitivity (robustness) of this network to the presence (absence) of each individual element. Accordingly, we introduce the notion of a path-degree of a vertex in terms of its corresponding incoming, outgoing and mediated paths, respectively. The pairwise disconnectivity index has been applied to the analysis of several regulatory networks from various organisms. The importance of an individual vertex or edge for the coherence of the network is determined by the particular position of the given element in the whole network.

**Conclusion:**

Our approach enables to evaluate the effect of removing each element (i.e., vertex, edge, or their combinations) from a network. The greatest potential value of this approach is its ability to systematically analyze the role of every element, as well as groups of elements, in a regulatory network.

## Background

Recent advances in graph theory have provided a new view on the topological design of different real-world networks [1-6]. Such systems exhibit small-world properties: They are surprisingly compact (i.e., their diameter is rather small) and display increased clustering features [7]. Moreover, they show a scale-free topology and follow a power-law type of the degree distribution: most components exhibit only one or two connections, but a few are involved in dozens and function as hubs, thereby providing networks with high robustness against random failures [1-3]. Various biological networks, such as metabolic or protein-protein interaction networks, show a scale-free topology [1,2,5] that emerges as a hallmark of modern systems biology.

However, by itself, the fact that a network has scale-free features is of limited practical use to biologists because power laws occur widely in nature and can have many different origins [8]. Currently, there is a gap between purely theoretical studies of the topology of large regulatory networks, on the one hand, and the practical traditions and interests of experimentalists, on the other hand. While the theoretical approaches emphasize the global characterization of regulatory systems as whole entities, experimental (even high-throughput) approaches usually focus on the role of distinct molecules and genes in regulation. There is a rather limited interface between them. Both approaches have not been integrated to study complex regulatory systems. To reconcile these apparently opposite views, one needs to combine 'general' with 'particular' aspects, as it is attempted by modern systems biology approaches, and translate rather abstract topological features of large systems into testable functional characteristics of individual components. So far, few such graph-theoretical characteristics have been explored for the analysis of biological networks [9-11], which are expected to have their particular properties.

There is a great need for approaches capable to quantitatively evaluate the importance of individual components in complex biological systems. Centrality analysis provides a valuable method for the structural, i.e. topological, analysis of biological networks. It allows to identify key elements within networks and to rank network elements such that experiments can be tailored to interesting candidates [10,11]. Local approaches such as the degree of a vertex (i.e., the number of its adjacent edges) help to find important molecules/genes which directly control many other molecules/genes, but fail to identify key regulators which are capable of affecting other molecules/genes in an indirect fashion. Other parameters, such as closeness and betweenness centrality, consider both local and distant connections within a network [9-12]. Closeness centrality evaluates how close a vertex (molecule/gene) is to all other vertices. Betweenness centrality measures how frequently a vertex appears on all shortest paths between two other vertices in a whole network [12-14]. Liu and colleagues [15] tested relationships between the phylogenetic profile of an enzyme and its topological importance in metabolic networks. They found that betweenness centrality is a good predictor of how many bacterial species have a particular enzyme. In contrast, the relationship with closeness centrality is much weaker or non-existent. This reflects the fact that the closeness centralities of a vertex and its immediate neighbors are rather similar and differ much less than their betweenness centralities. The representative power of betweenness centrality as a biologically relevant parameter was further confirmed in the topological analysis of mammalian networks of transcription factor genes: Among several topological characteristics tested, the betweenness centrality of individual transcription factor genes was found to be the most representative and relevant in regard to the biological significance of distinct elements [16]. In protein networks, betweenness centrality is rather helpful for identifying key connector proteins, i.e., bottlenecks, with particular functional and dynamic properties [17]. Betweenness centrality has been used to search for community structures in biological networks [12] and their hierarchical decomposition into subnetworks [18]. Thus, betweenness centrality has emerged as a promising measure of the biological significance of network elements.

Unfortunately, the approach based on the betweenness centrality suffers from some significant limitations due to the inherent nature of this parameter, which are finally becoming manifested in a restricted qualification for the analysis of regulatory networks. In the following we identify these limitations and propose with the pairwise disconnectivity index a new methodology that overcomes them. Subsequently, we apply the method to the analysis of various biological networks.

## Results

### Betweenness centrality and its limitations in analyzing regulatory networks

In regard to the needs of an analysis of regulatory networks there are two major disadvantages of betweenness centrality. Firstly, shortest paths are supposed to be the most important ones, which is a big oversimplification and misleading. The importance of a path is determined not so much by its length, i.e., the number of reactions, but rather by the integral efficiency of all these reactions. This efficiency depends on many instances, such as the concentrations of the participants, rate constants, etc. Longer paths can be faster and more efficient than shorter ones. For instance, in regulatory networks, the initiation of transcription and translation is typically governed by sets of specific factors. This increases the length of the corresponding paths, but drastically improves the efficiency and specificity of these processes. In a similar way, scaffold and adaptor proteins, which themselves are not enzymes, recruit downstream effectors in signaling pathways and enhance both the efficiency and specificity of signal propagation. Moreover, in most regulatory networks, like gene networks, an inherent problem is that the real length of edges is not defined at all. Each single edge commonly summarizes a set of events and describes the causal relations between genes. But this kind of abstraction does not say anything about the complexity and length of the corresponding processes. Thus, dealing with inconsistent semantics of the edges renders the definition of a shortest path in these networks highly problematic.

**Figure 1:**
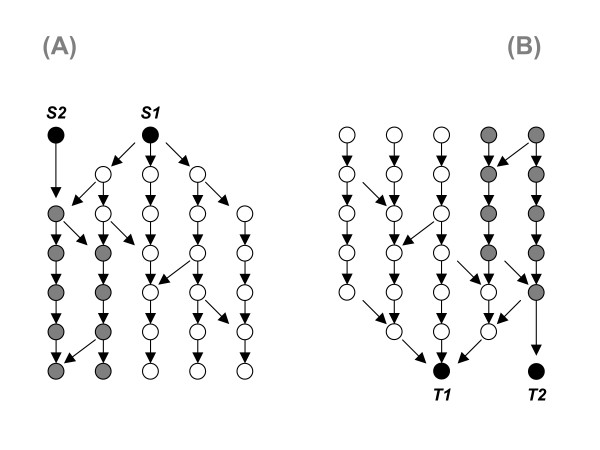
**Some vertices at the periphery of a regulatory network (the places where signals start or get their targets) can be rather significant.*****A***: The topological impact of start-point *S1 *is bigger than that of start-point *S2*. Both, white and gray vertices are on some path beginning in *S1*, while *S2 *is limited on the gray ones. ***B***: The topological significance of end-point *T1 *is bigger than that of end-point *T2 *because of being reachable from all gray and white vertices. However, in terms of betweenness centrality, all of them are attributed with zero values which fail to reflect the individual connectedness of such input/output elements within the whole network.

Secondly, betweenness centrality can be applied only to vertices that are between other ones. Peripheral vertices, i.e., vertices having either zero incoming or outgoing degree, are not considered. That immediately excludes many extracellular ligands, receptors, target molecules and genes from the analysis of a signaling network (Figure 1). Such components, however, directly respond to input-output functionality of the network and therefore are of key significance. Moreover, their individual topological significance in the network may vary in a wide range, as it can be seen when comparing the connectedness of the start-points S1 and S2, or end-points T1 and T2 in Figure 1. However, in terms of betweenness centrality, all of them are attributed with zero values which fail to reflect the individual connectedness of such input/output elements within the whole network.

We therefore developed the concept of the pairwise disconnectivity index as a new topological metric, which evaluates alternative though longer paths as well and can be used to characterize the topological significance of all individual elements in biological regulatory networks. The approach has some similarity to numerical parameters like *vertex-connectivity *or *edge-connectivity *used in graph theory to measure a graph's connectedness [19]. However, our method does not focus on how the removal of distinct elements breaks a given connected graph into disconnected pieces, like the algorithm of Girvan and Newman [12], though a network's disintegration can be considered as well. Instead, our aim was to find a parameter describing more moderate effects in a still connected network.

### Topological significance of individual elements in a regulatory network

In a directed graph *G*(*V*,*E*) representing a regulatory network, the vertices *v *∈ *V *denote biological entities, e.g., proteins, genes, or small molecules. Causal relationships between these entities are made up of directed edges *e *∈ *E*. We denote the *topological significance *of an individual element (vertex, edge or their combination) as how essential for all connections in the network this element is. To quantify this significance we suggest to measure how the elimination of such an element affects the number of connected ordered pairs of vertices. An ordered pair of vertices {*i*, *j*} ¦ i ≠ j and i, j ∈ V, is connected iff there is at least one path from vertex *i *to vertex *j *in *G*. Note, that the ordered pair {*i, j*} is different from {*j, i*} in a directed network. The more ordered pairs become disconnected upon the removal of vertex *v*, the higher is the topological significance of this vertex. We define the *pairwise disconnectivity index of vertex v*, *Dis*(*v*), as the fraction of those initially connected pairs of vertices in a network which become disconnected if vertex *v *is removed from the network

(1) $$Dis(v)=\frac{N_{0}-N_{-v}}{N_{0}}=1-\frac{N_{-v}}{N_{0}}$$

**Figure 2:**
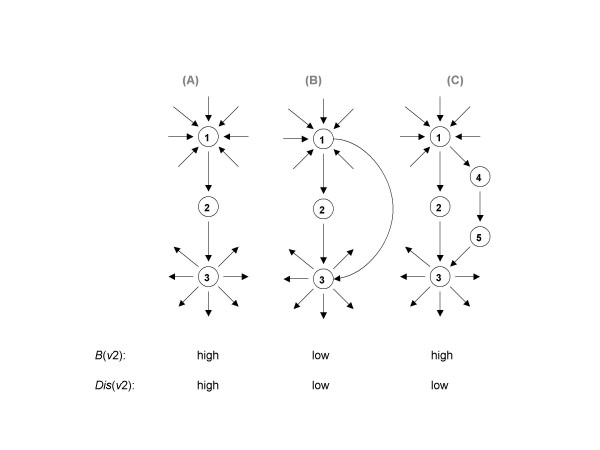
**The significance of a vertex is determined by its local and global environments in a network and can be better represented by a set of topological parameters.** In cases ***A***, ***B ***and ***C***, the degree of vertex 2 is the same. However, its betweenness centrality in ***A***, ***B ***and ***C ***is high, low and high, respectively, and the parwise disconnectivity index in ***A***, ***B ***and ***C ***is high, low and low, respectively. Thus, focusing on betweenness centrality may yield to misleading conclusions.

Here, *N*_0 _is the total number of ordered pairs of vertices in a network that are connected by at least one directed path of any length. It is supposed that *N*_0 _> 0, i.e., there exists at least one edge in the network that links two different vertices. *N*_-*v *_is the number of ordered pairs that are still connected after removing vertex *v *from the network, via alternative paths through other vertices (see vertex 2 in Figure 2B,C). However, the relation of *N*_-*v *_and *N*_0 _conveyed by *Dis*(*v*) immediately uncovers the fraction of connected ordered pairs whose communication essentially depends on vertex *v*. In the extreme case the removal of vertex *v *destroys all communication in a network resulting in *Dis*(*v*) = 1. In contrast, *Dis*(*v*) = 0 refers to a non-crucial vertex which is obviously not connected to any other vertex in a network.

**Figure 3:**
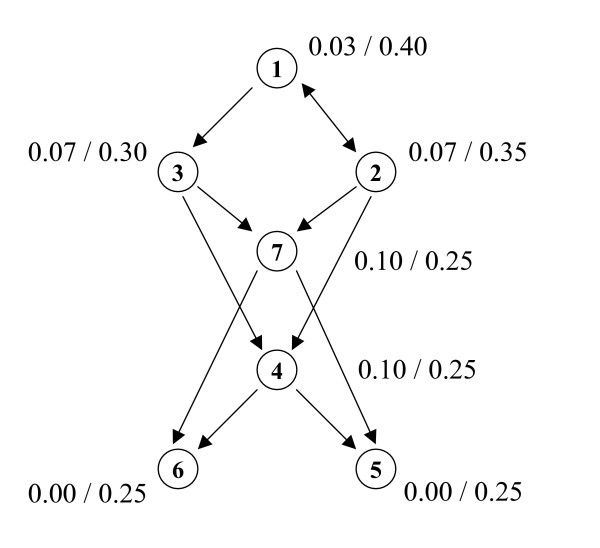
**Example for a network which demonstrates that betweenness centrality and pairwise disconnectivity index reflect different properties of a vertex.** While the vertices 4 and 7 are mediating many shortest paths, the removal of one of them does not cause a high damage to the existing connections because they provide alternative paths. In contrast, vertex 1 displays a modest betweenness centrality only but has the highest topological significance. The values of the betweenness centrality and the pairwise disconnectivity index of each vertex are indicated as *B*(*v*)/*Dis*(*v*), respectively.

The example presented in Figure 2 also illustrates the difference between the pairwise disconnectivity index and betweenness centrality. Vertex 2 is characterized with equally high (case A) or low (case B) values of both centralities, whereas they largely differ in case C (high betweenness centrality, but low pairwise disconnectivity index). The toy network in Figure 3 further illustrates that betweenness centrality and pairwise disconnectivity index reflect different properties of a vertex in a network. While the vertices 4 and 7 are mediating most of the shortest paths, thereby exhibiting a very high betweenness centrality value, these vertices show a rather low pairwise disconnectivity index since they provide alternative paths. In contrast, vertex 1 displays modest betweenness centrality but has a high topological significance according to its disconnectivity value (Figure 3). Thus, a vertex with high betweenness is not obligatorily topologically significant according to its disconnectivity value. It is only a clue for the fraction of short communication paths between reachable vertices which are provided due to the existence of a particular vertex.

Furthermore, the difference between the pairwise disconnectivity index and betweenness centrality becomes apparent when taking a closer look into the kind of reachable ordered pairs whose connection depends on vertex *v*. The complete set of those pairs, *N*_0 _- *N*_-*v*_, may include those which are connected by 1) paths that end at vertex *v*, 2) paths that start at vertex *v*, and 3) paths that go through vertex *v*. Other pairs cannot be affected, since they are connected via paths that do not contain any of the edges around vertex *v*. Accordingly, the pairwise disconnectivity index of vertex *v *can be represented as follows

(2) $$Dis(v)=\frac{N_{0}-N_{-v}}{N_{0}}=\frac{\sigma_{sv}+\sigma_{st}(v)+\sigma_{vt}}{N_{0}}$$

The term *σ*_*st*_(*v*) in Eq. 2 expresses the number of ordered pairs {*s*,*t*} ¦ s ≠ t ≠ v and s, t, v ∈ V that are exclusively linked through vertex *v*. Both, *σ*_*sv *_and *σ*_*vt *_involve *v *and represent the path-degree of vertex *v *in terms of all incoming and outgoing paths, respectively. Altogether, *σ*_*st*_(*v*) is not a trivial combination of *σ*_*sv *_and *σ*_*vt *_as Figure 2 shows: Vertex 2 is indeed crucial for connecting vertex 1 to vertex 3 in graph 2A. But in graphs 2B and 2C the same connection 1 → 3 does not depend on vertex 2 anymore, because of the parallel paths. However, vertex 2 still is essential for all paths that start or end in this vertex. The number of such ordered pairs associated with vertex 2, *σ*_*s*2 _and *σ*_2*t*_, does not change in the graphs 2A, 2B and 2C, thereby indicating the absence of a simple relationship between the values of *σ*_*sv*_, *σ*_*vt *_and *σ*_*st*_(*v*).

Often one wants to know how many connected pairs {*i*,*j*} depend on a particular vertex *v *while disregarding those kinds of pairs that involve the considered vertex, i.e. where *v ≠ i *and *v *≠ *j*. For example, when analyzing the role of a receptor for the indirect communication of extracellular ligands with transcription factors, communication paths that start or end at the receptor need not to be considered. The term *σ*_*st*_(*v*) in equation 2 exactly comprises this sort of essentiality and we define

(3) $$MDis(v)=\frac{\sigma_{st}(v)}{N_{0}}$$

as the *mediative disconnectivity index of a vertex v*. It immediately detects the fraction of connected ordered pairs of vertices different from *v *for whose reachability vertex *v *is necessary. While the pairwise disconnectivity index of vertex 2 in Figure 2A involves the pairs {*1,2*}, {*1,3*} and {*2,3*} it's mediative disconnectivity index reveals that vertex 2 is uniquely bridging the connection for {*1,3*}.

The mediative disconnectivity index of a vertex may exhibit some similarity to the beweenness centrality of the vertex. A path that uniquely connects two vertices *i *and *j *and is destroyed after removing another vertex *v *is always the shortest path between *i *and *j*. However, betweenness centrality considers all shortest paths and *MDis*(*v*) uncovers the cases where vertex *v *is the only link for a connected pair *i *and *j*. The principal difference between these parameters is due to their different sensitivity to the presence of parallel paths: betweenness centrality is insensitive to the presence of longer bypasses, whereas *MDis*(*v*) is very sensitive to that.

Vertex removal is a strong interference in a network because it simultaneously removes all incoming and outgoing edges of that vertex. One can also perturb a network by selectively knocking out a particular edge. This is a relatively gentle intervention which can simulate various normal and pathological situations in a regulatory network when all components are still present, but due to a mutation in one of them some of its reactions are specifically disabled while others are still working. That is particularly important when considering the fact that edges are a kind of abstraction and simplification, as discussed above. Thus, we declare an edge as topologically significant in the same way as a vertex: The higher the number of ordered pairs that become disconnected the higher the topological significance of an eliminated edge. To quantify this, we introduce the *pairwise disconnectivity index of an edge*, *Dis*(*e*), which is defined as

(4) $$Dis(e)=\frac{N_{0}-N_{-e}}{N_{0}}$$

Again, *N*_0 _is the number of ordered pairs of vertices connected by means of at least one directed path in the network. *N*_-*e *_is the number of such pairs after removing edge *e *from the network. The pairwise disconnectivity index of an edge ranges between 0 ≤ *Dis*(*e*) ≤ 1. In Figure 2A we previously argued the dependence of the communication of the ordered pair {*1,3*} on vertex 2. With the disconnectivity index of an edge it becomes clear that it is not necessary to remove vertex 2 itself in order to destroy the pair {*1,3*}. Moreover, a disorder of either the incoming or outgoing edge of vertex 2 is enough to compass the same effect.

### Topological significance of a group of elements in a regulatory network

Not all major functional breakdowns of a network can be explained due to the failure of one single element, but rather to the dysfunction of a subset of vertices or edges. The malfunctioning of this subset may disrupt a significant number of communication lines because parallel paths may be destroyed simultaneously. For example, in Fig. 2C the ordered pair {*1,3*} stays connected unless the vertices 2 and 4 or 2 and 5 are taken out together. As the generalization of Eq. 1 we define the *pairwise disconnectivity index of a group of vertices*, *W *⊆ *V*, as

(5) $$Dis(W)=\frac{N_{0}-N_{-W}}{N_{0}}=1-\frac{N_{-W}}{N_{0}}$$

with *N*_-*W *_representing the number of connected ordered pairs after removing the set of vertices *W*. Note that *Dis*(*W*) cannot be inferred directly from the disconnectivity indices of individual vertices in *W*. This is due to the presence of parallel paths in a network. For example, vertex 4 (or vertex 7) in Figure 3 features a rather low pairwise disconnectivity index. But as part of the group 'vertex 4 AND vertex 7' it causes the network to split into two distinct parts.

Finally, in analogy to Eq. 4 the general case of the removal of an individual edge is given by the *pairwise disconnectivity index of a group of edges*, *F *⊆ *E*, as defined in Eq. 6.

(6) $$Dis(F)=\frac{N_{0}-N_{-F}}{N_{0}}=1-\frac{N_{-F}}{N_{0}}$$

Here also, *Dis*(*F*) cannot be inferred directly from the disconnectivity indices of individual edges in *F*.

### Applying the pairwise disconnectivity index to the analysis of biological regulatory networks

In a topological analysis of several biological networks (one signal transduction network, two transcription regulation networks, and a neuronal connectivity network), we comparatively evaluated the pairwise disconnectivity index of the individual vertices with their betweenness centrality.

Transcription networks are displayed here as directed graphs, in which the nodes represent transcription factor genes and edges represent regulatory relationships between them, i.e., the transcriptional regulation of another transcription factor gene. We used the two best characterized transcription regulation networks from organisms of different kingdoms: a bacterium (*Escherichia coli*) [20] and a unicellular eukaryote (the yeast *Saccharomyces cerevisiae*) [21].

**Figure 4:**
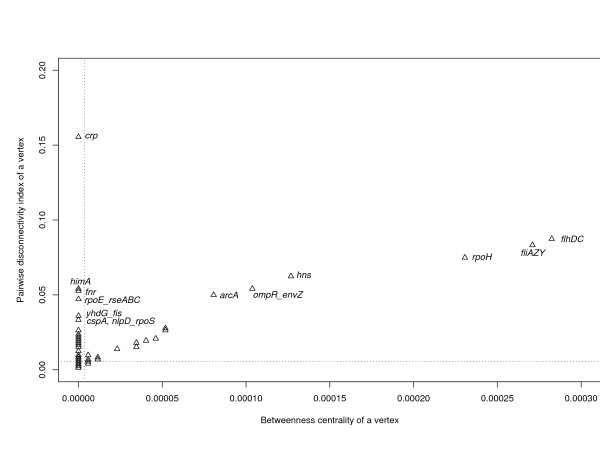
**Betweenness centrality, *B*(*v*), and the pairwise disconnectivity index, *Dis*(*v*), of all vertices in the *E. coli *transcriptional network.** The mean values of *B*(*v*) and *Dis*(*v*) are indicated with the vertical and horizontal dotted lines, respectively. Note that small values of *B*(*v*) and *Dis*(*v*) are attributed to most vertices in the network. The number of vertices in the network significantly exceeds the number of points in the plot: that is, many vertices having the same properties are represented by one point.

The *E. coli *transcriptional regulatory network consists of 423 vertices and 578 edges [20]. Small values of both *B*(*v*) and *Dis*(*v*) are attributed to most vertices in these networks, as it can be seen from the mean values of *B*(*v*) and *Dis*(*v*) (Figure 4). There is a strong positive correlation between the pairwise disconnectivity indices, *Dis*(*v*), and the corresponding values of betweenness centrality, *B*(*v*), for many genes, among them *arcA*, *ompR_envZ, hns, rpoH, fliAZY*, and *flhDC*. Their *Dis*(*v*) tends to be directly proportional to *B*(*v*) (Figure 4). However, we have found many exceptions to this trend. These are genes that exhibit low betweenness but relatively high disconnectivity: *crp*, *himA, fnr*, *rpoE_rseABC*, *yhdG_fis, cspA*, and *nipd_rpoS*. Gene *crp *shows the highest pairwise disconnectivity index. In the network analyzed, most of these genes display both nonzero incoming degree (*k*_*in *_> 0) and nonzero outgoing degree (*k*_*out *_> 0) and therefore have an internal position in the network. The protein product of gene *crp *is a well-characterized transcription activator triggered by cAMP and is responsible for regulating the expression of more than 100 genes in *E. coli *[22]. Moreover, genes *crp *(CRP), *fnr *(FNR) and *fis *(FIS) belong to the few global transcriptional regulators which are sufficient for directly modulating the expression of 51% of all genes in *E. coli *[23]. Betweenness centrality fails to identify them as topologically significant ones.

**Figure 5:**
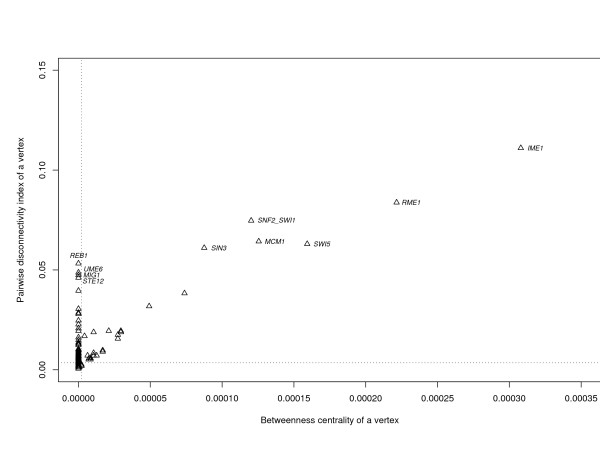
**Distribution of betweenness centrality, *B*(*v*), and the pairwise disconnectivity index, *Dis*(*v*), in the *S. cerevisiae *transcriptional network.** Small values of both *B*(*v*) and *Dis*(*v*) are attributed to most vertices, as it can be derived from the mean values of *B*(*v*) and *Dis*(*v*) (denoted with vertical and horizontal dotted lines, respectively).

Similar 'predictive weakness' of betweenness centrality is observed in the transcriptional network of *S. cerevisiae *(Figure 5). This network consists of 688 vertices and 1079 edges. Again, there is a strong positive correlation between the pairwise disconnectivity index of individual genes and the corresponding value of beweenness centrality. Such genes show a diagonal positioning on the plot. Small values of both *B*(*v*) and *Dis*(*v*) are attributed to most vertices in these networks, which thereby exhibit low topological significance. However, many genes with *B*(*v*) = 0, like *REB1, UME6, MIG1, STE12*, have high values of *Dis*(*v*) (Figure 5). In the network analyzed, all these genes exhibit no incoming degree (*k*_*in *_= 0) and are therefore positioned at the periphery of the network. The relatively large value of the pairwise disconnectivity index for these genes is in accordance with the roles they play in yeast. The product of gene *REB1 *(RNA polymerase I enhancer binding protein) is a DNA-binding protein that recognizes sites in both the enhancer and the promoter of rRNA transcription, as well as upstream of many genes transcribed by RNA polymerase II [24]. *REB1 *is essential for cell growth: its deletion mutant is inviable [25]. The other three genes of this group (*UME6*, *MIG1*, *STE12*) have important functions too, and deleting them solicits altered phenotypes, but is not lethal [26-31] [see Additional file 1]. Among those that have equally high values of the pairwise disconnectivity index and betweeness centrality, *MCM1 *is vital for the yeast cell [25,32]. Thus, at least one essential gene (*REB1*) was detected by the pairwise disconnectivity index, but this gene would have been missed by betweenness centrality because of its peripheral position in the network considered.

**Figure 6:**
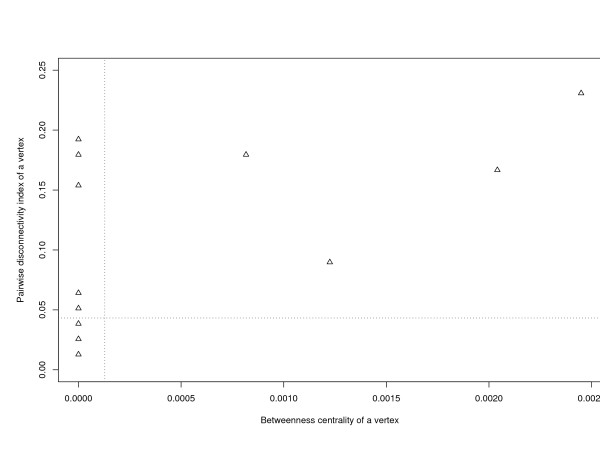
**Comparision of betweenness centrality, *B*(*v*), and the pairwise disconnectivity index, *Dis*(*v*), of individual vertices in the neuronal connectivity network of *C. elegans*.** Vertical and horizontal dotted lines stand for the mean values of *B*(*v*) and *Dis*(*v*), respectively.

We next analyzed the neuronal connectivity network of a simple multicellular organism, i.e. the nematode *Caenorhabditis elegans *[33]. Here, nodes represent neurons, and edges denote synaptic connections between the neurons. Each synaptic connection propagates a nerve impulse in one direction. This regulatory network includes 252 vertices and 509 directed edges. We found the same trend as in the transcription regulatory networks mentioned above: there are many vertices that display a low betweenness centrality combined with a high pairwise disconnectivity index (Figure 6): In contrast to the pairwise disconnectivity index, the betweenness centrality seems to underestimate the topological significance of some nodes, although we cannot comment here on their biological relevance since this is not documented.

**Figure 7:**
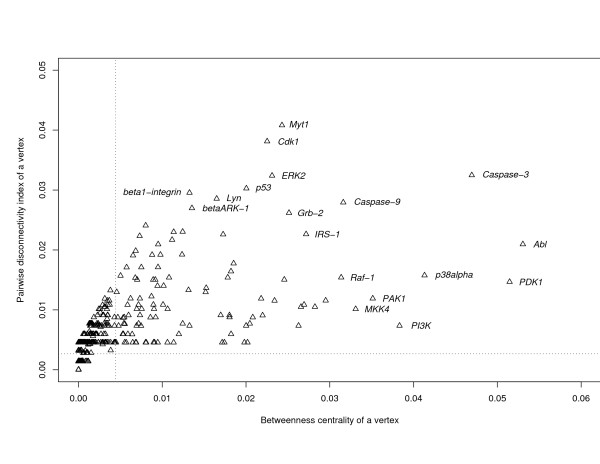
**Plot of betweenness centrality, *B*(*v*), and the pairwise disconnectivity index, *Dis*(*v*), for each vertex in the mammalian Toll-like receptor 4 signaling network. Mean values of *B*(*v*) and *Dis*(*v*) are drawn by vertical and horizontal dotted lines at a time.** Note that one point in the plot may represent many vertices having the same *B*(*v*) and *Dis*(*v*) properties.

The last example of regulatory networks refers to higher eukaryotes and is represented by the mammalian Toll-like receptor 4 (TLR4) signaling network. It controls a protective response of a host cell to a bacterial intervention and is important in activating the innate immunity [34,35]. The network consists of all signaling molecules that are reachable from the TLR4 receptor or from which the TLR4 receptor is reachable according to the contents of the TRANSPATH^® ^database on signal transduction [36]. It comprises of 742 vertices (molecules) and 1952 edges (reactions) and represents a genome-wide view at a level above the individual mammalian species. The contribution of individual vertices to sustaining the integrity of these paths varies significantly with the mean pairwise disconnectivity index of 0.0044 (Figure 7). That is, an average vertex is a crucial part of only 0.44% of the existing directed paths in the TLR4 network, thereby indicating the robust topological organization of the network. There are many molecules, like Myt1 (myelin transcription factor 1), Cdk1 (cyclin-dependent kinase 1), ERK2 (mitogen-activated protein kinase 2), p53 (tumor suppressor p53) and others, whose disconnectivity potential significantly exceeds this average level (Figure 7). Interestingly, all of them exhibit a lethal knockout effect in mice [see Additional file 1]. The pairwise disconnectivity index of vertices positively correlates with the corresponding values of betweenness centrality. In contrast to the transcriptional regulatory networks from *E. coli *and *S. cerevisiae *and the neuron connectivity network from *C. elegans *(Figures 4, 5, 6), the mammalian TLR4 network does have vertices which exhibit both low *B*(*v*) and high *Dis*(*v*) values. Moreover, the relationship of the pairwise disconnectivity index and betweenness centrality in the network is much more scattered. The bigger *B*(*v*) and *Dis*(*v*), the broader the scattering. Thus, there are many molecules which do not differ in their *B*(*v*) value, but significantly differ in their *Dis*(*v*) values and *vice versa*. Molecules Abl and PDK1 display the highest levels of *B*(*v*), but they are moderate in terms of *Dis*(*v*). That is, Abl and PDK1 are highly engaged in shortest-path communication in the network, but there are longer paths able to sustain the communication if either Abl or PDK1 is absent. In contrast to that, molecules Myt1, Cdk1 and ERK2 show the highest values of *Dis*(*v*), but they are moderate in terms of *B*(*v*) which means that although these proteins are not the most significant mediators of shortest-path communication in the TLR4 network they nevertheless provide the biggest impact on the topology of the network. Altogether, all these examples demonstrate that *Dis*(*v*) and *B*(*v*) represent different aspects of network organization.

**Figure 8:**
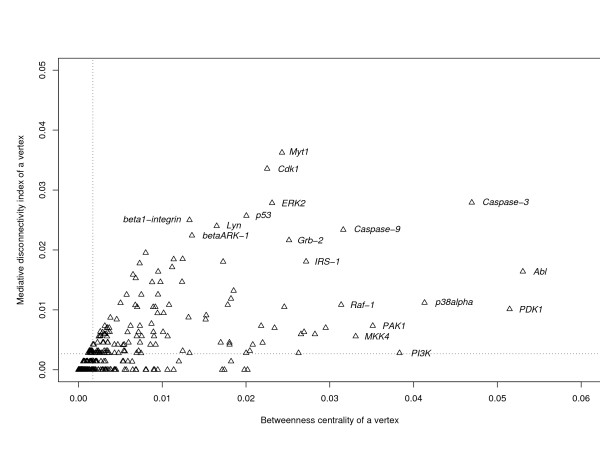
**Relationship of betweenness centrality, *B*(*v*), and the mediative disconnectivity index, *MDis*(*v*), of individual vertices in the mammalian TLR4 network.** The mean values of *B*(*v*) and *MDis*(*v*) are indicated with the vertical and horizontal dotted lines, respectively. Note that small values of *B*(*v*) and *MDis*(*v*) are attributed to most vertices in these networks.

In order to determine the most significant vertices that are conveying the communication between others, we calculated the mediative disconnectivity indices of all vertices, *MDis*(*v*), in the above mentioned networks and plotted them versus the corresponding values of betweenness centrality. The transcriptional networks from *E. coli *and *S. cerevisiae *and the neuron connectivity network from *C. elegans *show almost an ideal linear interdependence of *MDis*(*v*) and *B*(*v*) characterized by the correlation coefficients 0.99, 0.99 and 1.0, respectively [see Additional files 2, 3 and 4]. The corresponding mean values of *MDis*(*v*) are very small: 0.0008, 0.0006, and 0.004, respectively. Therefore, a small fraction of vertices are crucial as mediators of communication in these networks. Taken together, these networks, according to the present state of knowledge, appear to avoid significant parallelism of their paths and are relatively simply organized. In sharp contrast to that, the relationship of *MDis*(*v*) and *B*(*v*) in the mammalian TLR4 network is very scattered (Figure 8) and comparable with that of *Dis*(*v*) and *B*(*v*) (Figure 7). This network exhibits a higher complexity as compared to the previous ones. In that case, again, *MDis*(*v*) and *B*(*v*) characterize different aspects of network organization.

## Discussion

Robustness is a fundamental feature of complex evolvable systems and a ubiquitously observed property of biological systems [37,38]. Robustness means the maintenance of specific functionalities of the system, i.e., its homeostasis, against perturbations and it often requires the system to change its mode of operation in a flexible way [37,38]. That can be provided at the levels of the system structure, i.e., its topology, and/or the kinetics of multiple flows between its different parts. The structural reorganization via adding or removing of vertices and edges in the network plays the primary role and is decisive. Once established, these connections may be subject to fine-tuning by modulation of the corresponding reaction kinetics. We focused here on the topological aspects of regulation.

An extremely high topological robustness can be observed in a complete graph in which each vertex has direct links to all other vertices. In a complete graph with *V *vertices, all of them have the same, maximum possible degree *V-1*. Therefore, removing a vertex or an edge provides a minimal impact on the relationships in the remaining part of the network. Such an extreme robustness excludes any flexibility and does not satisfy the multiple functional tasks of biological regulation. That might be the reason for the fact that all studied biological networks are rather sparse [1-6]: that is, the density of their edges is very low.

Highly optimized tolerance (HOT) was recently introduced as a conceptual framework to link complexity to robustness as a trade-off of kind "robust yet fragile" [39-41]. By applying similar logic to the case of regulatory networks, we propose that the topology of regulatory networks must be evolutionarily adapted to optimally combine the necessary tolerance to noisy fluctuations (both internal and external) with the necessary sensitivity to some particular inputs. In other words, the design of regulatory networks must combine robust constructions, which sustain the homeostasis of a cell and an organism, with many different flexible constructions which may allow reorganization in response to particular inputs. Intracellular regulation is basically performed via varying the sets of molecules. Identification of the basic topology of a regulatory network and its associated trade-offs is essential for understanding the role of each particular element in regulation, as well as their faults and possible countermeasures – diseases and therapies, respectively.

The topological robustness closely relates to the number of alternative (i.e., parallel) paths in a regulatory system. Here, we introduced the pairwise disconnectivity index of a network's element to characterize how crucial it is in sustaining the communication within a network. This approach can be applied to the topological analysis of a regulatory network without making any preliminary simplifications like giving preferences to shortest paths, as it is made by the betweenness centrality approach. Shortest paths represent a small fraction of all paths in a network and even the notion of shortest paths in regulatory networks is questionable because of the 'fuzzy' semantics of edges in the corresponding graphs. This fuzziness is due to the typically undefined complexity of causal relationships between network elements. A causal link from gene *a *to gene *b*, that is displayed in a network by a single edge {*a, b*} and therefore appears to have length one, actually represents many steps at the level of transcription, RNA processing and splicing, transportation, translation, posttranslational modification, complex formation and so on. Thus, two edges can differ greatly in their elementary details. As a result, the path length is not a reliable variable for the analysis of such networks. The value of betweenness centrality of a given element, calculated on the basis of shortest paths passing this element, highly depends on the level of abstraction applied. Despite the very clear and attractive formalism of betweenness centrality [12,13], the practical usefulness of this measure in regard to cellular regulatory networks meets some problems due to the peculiarities of these networks.

To overcome the above mentioned shortcomings of betweenness centrality in regard to regulatory networks, all paths in the networks must be considered which is not feasible. Here, we introduced another strategy based on the fact that upon the removal of a given element some previously connected ordered pairs of vertices may become disconnected, thereby reducing the communication; this can be used to quantify the requirement of the element for the proper functioning of the whole network. Our approach emphasizes just the presence or the absence of causal links between vertices and does not rely on any assumptions concerning the meaning of these links. The pairwise disconnectivity index can be seen as a measure of topological non-redundancy of regulatory paths in a given network and, thus, as a measure of sensitivity of this network to the removal of each individual element.

The approach is rather similar to how biologists experimentally test the role of a given molecule or gene in a system of interest: the gene is knocked out or the molecule is inactivated by applying a proper inhibitor and so on. Accordingly, the evaluation of the effect of removing a vertex in a static context like a graph is the counterpart to knockout experiments performed in a lab. However, such virtual knockouts might simulate, to some extent, the corresponding wet experiments. They can be performed systematically for screening all vertices and edges in a network – which is not similarly efficiently feasible by experimental approaches. That opens up an attractive possibility to do targeted experimental verification for those elements for which a network analyses suggested topological significance. Finally, individual or groups of elements can be chosen as well for a static analysis enabling to focus on the particular context of the corresponding experiment. Altogether that might significantly contribute to a deeper understanding of network-wide interdependencies, causal relationships, and basic functional capabilities in cellular regulatory networks.

The approach has been applied to the analysis of several regulatory networks including the mammalian signal transduction TLR4 network, transcription regulatory networks from the bacteria *E. coli *and yeast *S. cerevisiae*, and the neuronal synaptic circuitry network from the nematode *C. elegans*. Different molecules, genes and neurons in these networks display a broad spectrum of pairwise disconnectivity index values, thus exhibiting a remarkable variability of the corresponding disconnectivity potentials. The impact of an individual vertex or edge is determined by its particular position in the whole network. This may be overlooked when using betweenness centrality, thereby underestimating the topological significance of some network elements.

In the *Dis*(*v*)-ranking of TLR4 network components (Figure 7), at least 3 out of the 4 top-ranking proteins (Cdk1, ERK2 and p53) are known as key signaling and transcription regulators in mammalian cells. All ten top-ranking genes (*Myt1, Cdk1, Caspase3, ERK2, p53, beta1-integrin, Lyn, Caspase9, betaARK-1 and Grb2*) are shown to be vital for living and developing of a mammalian organism: knockout of any of these genes causes a mutant phenotype 'inviable' [see Additional file 1]. This may serve as a benchmark that evidences the power of our method in identifying the biologically relevant key elements in regulatory networks.

By analyzing the interplay of *Dis*(*v*) and *B*(*v*), as well as *MDis*(*v*) and *B*(*v*), we have found notable difference in the organization of the mammalian TLR4 network as compared with the transcription networks from *E. coli *and yeast *S. cerevisiae*, and the neuronal synaptic network from *C. elegans *(Figures 4, 5, 6, 7, 8). The architecture of the TLR4 network exhibits a higher complexity. This might be due to various reasons: 1) the higher evolutional position of mammalian organisms, 2) the complexity of their intercellular organization, 3) differences in the organization of transcription and signal transduction networks which are adapted to different functional tasks, and 4) different completeness of our knowledge about these systems. To clarify the significance and the role of these reasons, new studies and additional analyses are necessary.

## Conclusion

A new topological metric, the pairwise disconnectivity index, has been proposed. The biological importance of the suggested approach relies on its capacity to quantitatively evaluate the topological significance of each element (i.e., vertex, edge, their groups and combinations) in the context of all other elements in a given regulatory network: that is how a given network can be regulated by means of its reorganization, i.e., removing an element and restoring the element. The approach enriches the set of tools available for the analysis of biological regulatory systems.

By applying the notion of the pairwise disconnectivity index to the analysis of several regulatory networks, we show that betweenness centrality and pairwise disconnectivitiy index represent different aspects of topological organization of regulatory networks. In general, there is a positive correlation between these approaches while evaluating the topological significance of individual elements in such networks. Nevertheless, in many cases the predictive power of betweenness centrality is really poor and is not biologically relevant. The pairwise disconnectivity index provides a much broader representation of topological peculiarities of individual elements in regulatory networks.

## Methods

### Network databases

Literature-based databases of experimentally verified direct relationships for *Escherichia coli *[20] and *Saccharomyces cerevisiae *[21] have been used where *E. coli *is available at [42] and *S. cerevisiae *at [43]. The neuronal synaptic circuitry network of *C. elegans *was obtained from the connectivity data for *Caenorhabditis elegans *available at [33]. The mammalian TLR4 network was retrieved from the contents of the TRANSPATH^® ^Professional database (release 7.3) on signal transduction [36] by searching for all elements that might be involved in communication with TLR4 receptor. That is, the network consists of all vertices that are reachable from TLR4 or from which TLR4 is reachable. In this network, molecules are represented at the level of "ortholog abstraction", at which all species-specific data that refer to mammalian molecules have been summarized to corresponding generic entries. Regulatory relationships between molecules and genes are displayed as semantic reactions of kind *X *→ *Y *where *X *and *Y *represent signal donors and acceptors, respectively. Such a semantic style is commonly used in the literature when describing regulatory pathways. The network [see Additional files 5 and 6] can be downloaded from [44].

Selected nodes in the yeast transcriptional and the TLR4 signaling network were checked for their viability using the BIOBASE Knowledge Library™ (BKL 1.2; BIOBASE GmbH, Wolfenbuettel, Germany) and the *Saccharomyces *Genome Database (Stanford Genomic Resources [45]).

### Graph analysis

#### Betweenness centrality

The values of betweenness centrality of vertices were computed by means of the network analysis software Pajek [46] as:

(8) $$B\left(v\right)=\frac{1}{\left(n-1)(n-2\right)}\sum_{s\neq t\neq v\in V}\frac{\delta_{st}\left(v\right)}{\delta_{st}}$$

Here, *δ*_*st *_is the total number of shortest paths between the nodes *s *and *t*, *δ*_*st*_(*v*) is those of them that pass through vertex *v*, and *n *is the number of vertices in the network. Note that the above definition represents the normalized betweenness centrality.

#### Pairwise disconnectivity index

The main idea of the pairwise disconnectivity index of a vertex, edge, for a group of vertices or edges is to compare the number of ordered pairs of vertices that are reachable in a graph before and after removing a vertex, edge and so on. Therefore, counting the number of ordered pairs is the essential part of any approach to determine the pairwise disconnectivity index. Various algorithms might be used for this purpose as for example depth-first (breadth-first) search or Dijkstra's shortest paths algorithm. For the analyses described here, we have developed a tool that uses a modified depth-first search to efficiently calculate the pairwise disconnectivity indices. To estimate the index for a vertex or edge, the implemented algorithm does not exceed *Θ*(*V^2^*). The program available at [47].

### Statistical analysis

Besides the already mentioned software, parts of the statistical analysis have been accomplished with support of the R project for statistical computing [48].

## Authors' contributions

APP developed the concept of pairwise disconnectivity indices, conceived of the study, analyzed and interpreted the data and drafted the manuscript. BG carried out the programming, performed the statistical analysis and drafted the manuscript. EW participated in the coordination of the study, helped to draft the manuscript and gave final approval of the version to be published. All authors read and approved the final manuscript.

## Supplementary Material

Additional file 1 Data on gene knockouts and their biological effects for the *Dis*(*v*)-top-ranking elements in the networks of *E. coli*, yeast and mammalian TLR4. Click here for file

Additional file 2 Relationship of betweenness centrality, *B*(*v*), and the mediative disconnectivity index, *MDis*(*v*), of individual vertices in *E. coli*. Click here for file

Additional file 3 Relationship of betweenness centrality, *B*(*v*), and the mediative disconnectivity index, *MDis*(*v*), of individual vertices *S. cerevisiae*. Click here for file

Additional file 4 Relationship of betweenness centrality, *B*(*v*), and the mediative disconnectivity index, *MDis*(*v*), of individual vertices in *C. elegans*. Click here for file

Additional file 5 The contents of the mammalian TLR4 network. Click here for file

Additional file 6 The contents of the mammalian TLR4 network. Click here for file
