# Supplementary material for: The pairwise disconnectivity index as a new metric for the topological analysis of regulatory networks
Source: BMC Bioinformatics. 2008 May 2;9:227. doi: 10.1186/1471-2105-9-227 (PMC2396639; doi:10.1186/1471-2105-9-227)
Supplement: Additional file 1 — Data on gene knockouts and their biological effects for the Dis(v)-top-ranking elements in the networks of E. coli, yeast and mammalian TLR4. [file 1471-2105-9-227-S1.pdf]

Network	Element	Dis(v)	Knockout effect	References
E.coli	crp	0.1553	slow-growth phenotype	43
E.coli	fnr	0.0527	phenotypically silent	43
E.coli	himA	0.0541	defective in phage lambda integration	44
E.coli	rpoE_rseABC	0.0471	enhanced expression of the superoxide sensor SoxR	45
E.coli	arcA	0.0499	increased aerobic TCA cycle activity	43
Yeast	REB1	0.0532	inviable; lethal	25, 46
Yeast	UME6	0.0487	viable; growth rate abnormal; lethal	25, 27, 47, 48, 50
Yeast	MIG1	0.0474	viable; altered genetic expression; Lag phase growth abnormal	25, 50, 51
Yeast	STE12	0.0461	viable; growth rate abnormal; filamentous growth abnormal	25, 52, 53
Yeast	IME1	0.1110	defective meiosis	54
Yeast	RME1	0.0838	viable; invasive growth abnormal; sexual sporulation abnormal	25, 55, 56
Yeast	SNF2/SWI2	0.0747	defective meiosis; growth rate abnormal; recombination defective	82, 83, 84
Yeast	SWI5	0.0629	viable; sodium sensitive; altered sensitivity to drugs or other compounds	25, 57
Yeast	MCM1	0.0643	inviable; lethal	25, 58
Yeast	SIN3	0.0610	viable; sexual sporulation abnormal; altered sensitivity to drugs or other compounds	25, 59, 60, 61
TLR4	Myt1	0.0408	inviable; Myt1(-/-) animals die postnatally	62
TLR4	Cdk1	0.0381	inviable	63, 64
TLR4	p53	0.0303	inviable	65, 66, 67
TLR4	Caspase3	0.0325	inviable	68, 69
TLR4	ERK2	0.0324	embryo-fetus inviable	70
TLR4	beta1-integrin	0.0295	embryo-fetus inviable	71, 72, 73, 74, 75
TLR4	Lyn	0.0285	Lyn-/- B cells are hyper-responsive to transmembrane signals; high levels of autoantibody production and glomerulonephritis	76, 77
TLR4	betaARK-1	0.0277	embryo-fetus inviable; neonate inviable	78, 79, 80
TLR4	Caspase9	0.0279	inviable	68, 69
TLR4	Grb-2	0.0262	neonate inviable	81

Table 1: Gene knockouts and their biological effects

References

25. Giaever G, Chu AM, Ni L, Connelly C, Riles L, Véronneau S, Dow S, Lucau-Danila A, Anderson K, André B, Arkin AP, Astromoff A, El-Bakkoury M, Bangham R, Benito R, Brachat S, Campanaro S, Curtiss M, Davis K, Deutschbauer A, Entian KD, Flaherty P, Foury F, Garfinkel DJ, Gerstein M, Gotte D, Güldener U, Hegemann JH, Hempel S, Herman Z, Jaramillo DF, Kelly DE, Kelly SL, Kötter P, LaBonte D, Lamb DC, Lan N, Liang H, Liao H, Liu L, Luo C, Lussier M, Mao R, Menard P, Ooi SL, Revuelta JL, Roberts CJ, Rose M, Ross-Macdonald P, Scherens B, Schimmack G, Shafer B, Shoemaker DD, Sookhai-Mahadeo S, Storms RK, Strathern JN, Valle G, Voet M, Volckaert G, Wang CY, Ward TR, Wilhelmy J, Winzeler EA, Yang Y, Yen G, Youngman E, Yu K, Bussey H, Boeke JD, Snyder M, Philippsen P, Davis RW, Johnston M: **Functional profiling of the Saccharomyces cerevisiae genome.** *Nature* 2002, 418: 387-391

27. Steber CM, Esposito RE: **UME6 is a central component of a developmental regulatory switch controlling meiosis-specific gene expression.** *Proc Natl Acad Sci USA* 1995, 92: 12490-12494

43. Perrenoud A, Sauer, U: **Impact of global transcriptional regulation by ArcA, ArcB, Cra, Crp, Cya, Fnr, and Mlc on glucose catabolism in Escherichia coli.** *J Bacteriol* 2005, 187: 3171-3179

44. Miller HI, Nash HA: **Direct role of the himA gene product in phage lambda integration.** *Nature* 1981 Apr 9;290(5806):523-6

45. Koo MS, Lee JH, Rah SY, Yeo WS, Lee JW, Lee KL, Koh YS, Kang SO, Roe JH: **A reducing system of the superoxide sensor SoxR in Escherichia coli.** *EMBO J* 2003, 22: 2614-2622

46. Liaw PC, Brandl CJ: **Defining the sequence specificity of the Saccharomyces cerevisiae DNA binding protein REB1p by selecting binding sites from random-sequence oligonucleotides.** *Yeast* 1994, 10: 771-787

47. Messenguy F, Vierendeels F, Scherens B, Dubois E: **In Saccharomyces cerevisiae, expression of arginine catabolic genes CAR1 and CAR2 in response to exogenous nitrogen availability is mediated by the Ume6 (CargRI)-Sin3 (CargRII)-Rpd3 (CargRIII) complex.** *J Bacteriol* 2000, 182: 3158-3164

48. Suzuki C, Hori Y, Kashiwagi Y: **Screening and characterization of transposon-insertion mutants in a pseudohyphal strain of Saccharomyces cerevisiae.** *Yeast* 2003, 20: 407-415

49. Goldmark JP, Fazzio TG, Estep PW, Church GM, Tsukiyama T: **The Isw2 chromatin remodeling complex represses early meiotic genes upon recruitment by Ume6p.** *Cell* 2000, 103: 423-433

50. Sarma NJ, Haley TM, Barbara KE, Buford TD, Willis KA, Santangelo GM: **Glucose-responsive regulators of gene expression in Saccharomyces cerevisiae function at the nuclear periphery via a reverse recruitment mechanism.** *Genetics* 2007, 175: 1127-1135

51. Klein CJ, Rasmussen JJ, Rønnow B, Olsson L, Nielsen J: **Investigation of the impact of MIG1 and MIG2 on the physiology of Saccharomyces cerevisiae.** *J Biotechnol* 1999, 68: 197-212

52. Niedenthal R, Riles L, Güldener U, Klein S, Johnston M, Hegemann JH: **Systematic analysis of S. cerevisiae chromosome VIII genes.** *Yeast* 1999, 15: 1775-1796

53. Lorenz MC, Cutler NS, Heitman J: **Characterization of alcohol-induced filamentous growth in Saccharomyces cerevisiae.** *Mol Biol Cell* 2000, 11: 183-199

54. Kassir Y, Granot D, Simchen G: **IME1, a positive regulator gene of meiosis in S. cerevisiae.** *Cell* 1988, 52: 853-862

55. van Dyk D, Hansson G, Pretorius IS, Bauer FF: **Cellular differentiation in response to nutrient availability: The repressor of meiosis, Rme1p, positively regulates invasive growth in Saccharomyces cerevisiae.** *Genetics* 2003, 165: 1045-1058

56. Covitz PA, Herskowitz I, Mitchell AP: **The yeast RME1 gene encodes a putative zinc finger protein that is directly repressed by a1-alpha 2.** *Genes Dev* 1991, 5: 1982-1989

57. Butcher RA, Schreiber SL: **Identification of Ald6p as the target of a class of small-molecule suppressors of FK506 and their use in network dissection.** *Proc Natl Acad Sci USA* 2004, 101: 7868-7873

58. Althoefer H, Schleiffer A, Wassmann K, Nordheim A, Ammerer G: **Mcm1 is required to coordinate G2-specific transcription in Saccharomyces cerevisiae.** *Mol Cell Biol* 1995, 15: 5917-5928

59. Vidal M, Strich R, Esposito RE, Gaber RF: **RPD1 (SIN3/UME4) is required for maximal activation and repression of diverse yeast genes.** *Mol Cell Biol* 1991, 11: 6306-6316

60. Jazayeri A, McAinsh AD, Jackson SP: **Saccharomyces cerevisiae Sin3p facilitates DNA double-strand break repair.** *Proc Natl Acad Sci USA* 2004, 101: 1644-1649

61. Lockshon D, Surface LE, Kerr EO, Kaeberlein M, Kennedy BK: **The sensitivity of yeast mutants to oleic acid implicates the peroxisome and other processes in membrane function.** *Genetics* 2007, 175: 77-91

62. Wang S, Zhang J, Zhao A, Hipkens S, Magnuson MA, Gu G: **Loss of Myt1 function partially compromises endocrine islet cell differentiation and pancreatic physiological function in the mouse.** *Mech Dev* 2007, 124: 898-910

63. Bashir T, Pagano M: **Cdk1: the dominant sibling of Cdk2.** *Nature Cell Biol* 2005, 7: 779 - 781

64. Santamaría D, Barrière C, Cerqueira A, Hunt S, Tardy C, Newton K, Cáceres JF, Dubus P, Malumbres M, Barbacid M: **Cdk1 is sufficient to drive the mammalian cell cycle.** *Nature* 2007, 448: 811-815

65. Perkins SN, Hursting SD, Haines DC, James SJ, Miller BJ, Phang JM: **Chemoprevention of spontaneous tumorigenesis in nullizygous p53-deficient mice by dehydroepiandrosterone and its analog 16alpha-fluoro-5-androsten-17-one.** *Carcinogenesis* 1997, 18: 989-994

66. Johnsen AK, France J, Nagy N, Askew D, Abdul-Karim FW, Gerson SL, Sy MS, Harding CV: **Systemic deficits in transporter for antigen presentation (TAP)-1 or proteasome subunit LMP2 have little or no effect on tumor incidence.** *Int J Cancer* 2001, 91: 366-372

67. van Meyel DJ, Sanchez-Sweatman OH, Kerkvliet N, Stitt L, Ramsay DA, Khokha R, Chambers AF, Cairncross JG: **Genetic background influences timing, morphology and dissemination of lymphomas in p53-deficient mice.** *Int J Oncol* 1998, 13: 917-922

68. Colussi PA, Kumar S: **Targeted disruption of caspase genes in mice: What they tell us about the functions of individual caspases in apoptosis.** *Immunol Cell Biol* 1999, 77: 58?63

69. Kuida K, Haydar TF, Kuan CY, Gu Y, Taya C, Karasuyama H, Su MS, Rakic P, Flavell RA: **Reduced Apoptosis and Cytochrome c?Mediated Caspase Activation in Mice Lacking Caspase 9.** *Cell* 1998, 94: 325-337

70. Hatano N, Mori Y, Oh-hora M, Kosugi A, Fujikawa T, Nakai N, Niwa H, Miyazaki J, Hamaoka T, Ogata M: **Essential role for ERK2 mitogen-activated protein kinase in placental development.** *Genes Cells* 2003, 8: 847-856

71. Aumailley M, Pesch M, Tunggal L, Gaill F, Fässler R: **Altered synthesis of laminin 1 and absence of basement membrane component deposition in (beta)1 integrin-deficient embryoid bodies.** *J Cell Sci* 2000, 113: 259-268

72. Damsky Ch, Schick SF, Klimanskaya I, Stephens L, Zhou Y, Fisher S: **Adhesive interactions in peri-implantation morphogenesis and placentation.** *Reprod Toxicol* 1997, 11: 367-375

73. Stephens LE, Sutherland AE, Klimanskaya IV, Andrieux A, Meneses J, Pedersen RA, Damsky CH: **Deletion of beta 1 integrins in mice results in inner cell mass failure and peri-implantation lethality.** *Genes Dev* 1995, 9: 1883-1895

74. Rohwedel J, Guan K, Zuschratter W, Jin S, Ahnert-Hilger G, Fürst D, Fässler R, Wobus AM: **Loss of beta1 integrin function results in a retardation of myogenic, but an acceleration of neuronal, differentiation of embryonic stem cells in vitro.** *Dev Biol* 1998, 201: 167-184

75. Baudoin C, Goumans MJ, Mummery C, Sonnenberg A: **Knockout and knockin of the beta1 exon D define distinct roles for integrin splice variants in heart function and embryonic development.** *Genes Dev* 1998, 12: 1202-1216

76. Hasegawa M, Fujimoto M, Poe JC, Steeber DA, Lowell CA, Tedder TF: **A CD19-dependent signaling pathway regulates autoimmunity in Lyn-deficient mice.** *J Immunol* 2001, 167: 2469-2478

77. Yu CC, Yen TS, Lowell CA, DeFranco AL: **Lupus-like kidney disease in mice deficient in the Src family tyrosine kinases Lyn and Fyn.** *Curr Biol* 2001, 11: 34-38

78. Peppel K, Boekhoff I, McDonald P, Breer H, Caron MG, Lefkowitz RJ: **G protein-coupled receptor kinase 3 (GRK3) gene disruption leads to loss of odorant receptor desensitization.** *J Biol Chem* 1997, 272: 25425-25428

79. Jaber M, Koch WJ, Rockman H, Smith B, Bond RA, Sulik KK, Ross J Jr, Lefkowitz RJ, Caron MG, Giros B: **Essential role of beta-adrenergic receptor kinase 1 in cardiac development and function.** *Proc Natl Acad Sci USA* 1996, 93: 12974-12979

80. Rockman HA, Choi DJ, Akhter SA, Jaber M, Giros B, Lefkowitz RJ, Caron MG, Koch WJ: **Control of myocardial contractile function by the level of beta-adrenergic receptor kinase 1 in gene-targeted mice.** *J Biol Chem* 1998, 273: 18180-18184

81. Saxton TM, Cheng AM, Ong SH, Lu Y, Sakai R, Cross JC, Pawson T: **Gene dosage-dependent functions for phosphotyrosine-Grb2 signaling during mammalian tissue morphogenesis.** *Curr Biol* 2001, 11: 662-670

82. Marston AL, Tham WH, Shah H, Amon A: **A genome-wide screen identifies genes required for centromeric cohesion.** *Science* 2004, 303: 1367-1370

83. Laurent BC, Treitel MA, Carlson M: **Functional interdependence of the yeast SNF2, SNF5, and SNF6 proteins in transcriptional activation.** *Proc Natl Acad Sci USA* 1991, 88: 2687-2691

84. Dror V, Winston F: **The Swi/Snf chromatin remodeling complex is required for ribosomal DNA and telomeric silencing in Saccharomyces cerevisiae.** *Mol Cell Biol* 2004, 24: 8227-8235
